# Supplementary material for: A Comprehensive Assessment of Ultraviolet-Radiation-Induced Mutations in Flammulina filiformis Using Whole-Genome Resequencing
Source: J Fungi (Basel). 2024 Mar 20;10(3):228. doi: 10.3390/jof10030228 (PMC10971301; doi:10.3390/jof10030228)
Supplement: Supplementary file 1 [file jof-10-00228-s001.zip › Supplementary Material S8/KEGG annotation/out/64381550635650.os/KO/out_map/map05416.html]

KEGG PATHWAY: Viral myocarditis - Reference pathway


|  |  |
| --- | --- |
| **Viral myocarditis - Reference pathway** |  |

[
Pathway menu
| Organism menu
| Pathway entry
| Show description
| User data mapping
]

Myocarditis is a cardiac disease associated with inflammation and injury of the myocardium. It results from various etiologies, both noninfectious and infectious, but coxsackievirus B3 (CVB3) is still considered the dominant etiological agent. Myocarditis may be caused by direct cytopathic effects of virus, a pathologic immune response to persistent virus, or autoimmunity triggered by the viral infection. The virus enters the myocyte through internalization of the coxsackie-adenoviral receptor (CAR) and its coreceptor, decay-accelerating factor (DAF). Viral proteases cleave various proteins in the host cell. One example is viral protease 2A, which cleaves eukaryote initiation factor 4G (eIF4G) and the dystrophin protein, resulting in a complete shutdown of cap-dependent RNA translation and cytoskeletal destruction in infected cardiomyocytes, respectively. CVB3 also cleaves the member of the Bcl-2 family Bid, leading to apoptosis. CVB3 infection also induces the cleavage of cyclin D protein through a proteasome-dependent pathway, leading to the host cell-growth arrest. Viral infection and necrosis of myocytes may lead to the release of intracellular antigens, resulting in activation of self-reactive T cells. CVB infection is a significant cause of dilated cardiomyopathy (DCM) as well as myocarditis. Epidemiologically, myocarditis underlies a significant portion of patients with DCM.

|  |  |
| --- | --- |
| Reference pathway | 100% |
